# VIX constant maturity futures trading strategy: *A walk-forward machine learning study*

**DOI:** 10.1371/journal.pone.0302289

**Published:** 2024-04-19

**Authors:** Sangyuan Wang, Keran Li, Yaling Liu, Yijun Chen, Xianbo Tang

**Affiliations:** 1 Southwestern University of Finance and Economics, Chengdu, Sichuan, China; 2 The University of Sydney, Sydney, New South Wales, Australia; Institute for Economic Forecasting, Romanian Academy, ROMANIA

## Abstract

This study employs seven advanced machine learning approaches to conduct numerical predictions of the next-day returns of VIX constant-maturity futures (VIX CMFs) using the term structure information derived from VIX CMFs. Based on precise numerical predictions, this study proposes a new Constrained-Mean-Variance Portfolio Optimization (C-MVO) trading strategy and tests it against a benchmark long-short trading strategy to evaluate the profitability of the machine learning numerical predictions. This study applies three unique feature sets, each incrementally incorporating the VIX CMFs’ term structure features, to individually examine the predictive ability of the seven machine learning models and their backtesting performance. Over a comprehensive 11-year period, the experiment adheres to a strict walk-forward expanding-window methodology for both training and backtesting. The predictive and backtesting results show that four of the seven machine learning models attain a prediction information ratio greater than 0.02, with an average prediction information ratio of 0.037. This result suggests that the VIX CMFs term structure features have predictive power for the next-day returns of VIX CMFs. Moreover, the average C-MVO information ratio is 0.623, and the long-short strategy information ratio is 0.404. This increase in the information ratio under the C-MVO strategy validates the effectiveness of the machine learning models and the C-MVO strategy.

## 1. Introduction

VIX, often called the “investor fear gauge,” has been a pivotal index in the financial market since its inception in the early 1990s. Since 2003, the VIX has reflected the 30-day Standard & Poor’s 500 (S&P 500) expected volatility implied in various call and put S&P 500 options [1, 2]. The VIX is not directly tradeable. Instruments such as VIX futures, options, and exchange-traded notes (ETN) are essential tools for trading the VIX. With investors’ growing interest in the VIX, S&P introduced the VIX futures index series in 2005. This series replicates VIX CMFs by systematically rolling and combining futures contracts with different maturities. Specifically, the VIX short-term futures index (SPVXSP) maintains a fixed 1-month constant maturity; VIX two-month futures index (SPVIX2ME) maintains a constant 2-month maturity; and several others include three-month VIX futures-(SPVIX3ME), four-month VIX CMFs (SPVIX4ME), five-month VIX CMFs (SPVIXMP), and six-month VIX CMFs (SPVIX6ME). Table 1 presents the descriptive statistics of the six CMFs and Table 2 describes the correlations between the six VIX CMFs.

**Table 1:** Descriptive statistics of VIX CMFs.

	SPVXSP	SPVIX2ME	SPVIX3ME	SPVIX4ME	SPVIXMP	SPVIX6ME
mean	-0.0014	-0.0007	-0.0004	-0.0003	-0.0002	-0.0001
std	0.0466	0.0351	0.0280	0.0240	0.0217	0.0199
min	-0.2596	-0.2098	-0.1875	-0.1773	-0.1552	-0.1301
25%	-0.0248	-0.0185	-0.0147	-0.0128	-0.0113	-0.0102
50%	-0.0064	-0.0036	-0.0027	-0.0023	-0.0017	-0.0013
75%	0.0161	0.0134	0.0109	0.0099	0.0090	0.0082
max	0.9610	0.7196	0.4647	0.2962	0.2654	0.2356

**Table 2:** Correlations of VIX CMFs.

	SPVXSP	SPVIX2ME	SPVIX3ME	SPVIX4ME	SPVIXMP	SPVIX6ME
SPVXSP	1.000	0.974	0.952	0.920	0.907	0.890
SPVIX2ME	0.974	1.000	0.981	0.954	0.941	0.925
SPVIX3ME	0.952	0.981	1.000	0.986	0.975	0.958
SPVIX4ME	0.920	0.954	0.986	1.000	0.993	0.978
SPVIXMP	0.907	0.941	0.975	0.993	1.000	0.994
SPVIX6ME	0.890	0.925	0.958	0.978	0.994	1.000

The VIX term structures describe the relationship between VIX futures contracts with varying expiration dates. The term structure is in contango when VIX futures with longer maturities have higher prices than futures with shorter maturities. Backwardation occurs when the prices of VIX futures with a shorter time to expiration exceed those with longer maturities. Previous empirical analyses robustly indicate a dominant contango within the VIX future term structure. This phenomenon can be partially explained by volatility risk premium theory and Johnson’s (2017) work, which divides VIX term structure into two fundamental components: conditional volatility expectations and risk premiums [3, 4]. It becomes apparent that investors normally require a larger risk premium for further VIX options than for shorter-maturity VIX options, except during certain financial crises. Notably, the VIX futures term structure holds predictive ability when anomalies in the term structure revert to the classical shape, given the strong mean-reverting property of VIX futures, as Avellaneda and Papanicolaou illustrate [5].

Numerous studies, including those by Latane and Rendleman (1976), Szakmary and Ors (2000), Busch et al. (2011), and Byun and Kim (2013), demonstrate that IV-related derivatives, such as VIX and their term structures, possess strong predictive abilities for future volatility [6–9]. However, a significant challenge exists: the IV and VIX are not directly tradable instruments. This limitation means that traders cannot leverage these insights to execute trades or to profit directly, despite their theoretical value in forecasting market movements. This gap highlights the need to translate these theoretical insights into practical trading strategies using tradable instruments linked to the IV and VIX, such as VIX CMFs, to effectively harness their predictive values. Meanwhile, the predictive ability of VIX tradable derivative term structures remains unknown. However, few studies focus on the predictability of tradable VIX derivatives, such as VIX futures, and the construction of trading strategies. For example, early studies by Konstantinidi et al. (2008), Degiannakis (2008), and Asensio(2013) discover the limited predictability of VIX futures with weak performance in trading strategies [10–12]. With the boom in machine learning approaches, studies such as Guizzardi and Palladini (2019), Vrontos (2021), and Avellaneda et al. (2021) apply machine learning approaches to predict VIX derivatives and construct trading strategies [13–15]. However, rather than providing precise numerical predictions, previous studies focused on either interval or directional forecasting of the VIX. We clearly see a gap in terms of whether accurate numerical predictions be made for tradable VIX derivatives using the latest machine learning models, and if these predictions facilitate the construction of a profitable trading strategy.

Therefore, this study tries to address the challenge of tradability of underlying assets and bridge the gap in numerical predictions using machine learning approaches, with the aim of discovering VIX tradable derivatives’ term structure predictive ability and developing profitable trading strategies. We employ seven machine learning models, including neural-based network models, tree-based models, and vanilla models, to explore the predictive ability of VIX CMFs term structure for the next-day returns of VIX CMFs. Furthermore, complemented by numerical predictions, we introduce our newly proposed C-MVO strategy along with a benchmark long-short strategy to further investigate the potential for additional profits from such predictions.

### 1.1 Contributions

The key contributions of this study are at least fourfold. First, based on the VIX CMFs decomposition methods proposed by Avellaneda et al. (2021) [15], we further examine the predictive ability of VIX CMFs term structures, specifically *μ*_*t*_ and △*roll*. These features are then integrated into machine learning models as novel predictors. Our findings indicate that *μ*_*t*_ and △*roll* are highly informative in predicting the next-day returns of VIX CMFs and can yield decent portfolio performance, as evidenced by our backtesting analysis. Second, it differs from previous studies that perform either interval or directional predictions. Our study employs a wide range of machine-learning models to generate numerical predictions for the next-day returns of VIX CMFs. Such numerical predictions enable the development of trading strategies with more precise risk management and specific designs. In our case, numerical predictions allow us to use a mean-variance optimization structure to construct trading strategies. Third, we present our newly proposed C-MVO trading strategy, which integrates numerical predictions for next-day returns and outperforms the benchmark long-short trading strategy in terms of annualized returns and information ratios. Finally, we employ multiple machine learning approaches and two trading strategies, adhering to strict walk-forward model training and backtesting procedures. This implementation provides researchers and practitioners with a reliable and thorough framework for integrating machine-learning approaches into quantitative financial trading.

The remainder of this paper is organized as follows. Section 2 presents a brief literature review, and Section 3 details the research methodology. Section 4 describes the data preparation and experiment design. Section 5 analyzes and discusses the results. Section 7 concludes the article, and the supporting information includes backtesting results for all machine learning models under all feature sets.

## 2 Literature review

### 2.1 Predictive ability of implied volatility (IV), VIX, and their term structures

Previous research thoroughly investigated the predictive ability of implied volatility (IV). Early research by Latane and Rendleman (1976) demonstrates that Black-Scholes call option IVs exhibit superior predictive performance for the future volatility of the S & P 500 index [6]. Szakmary (2000) confirms that IVs derived from 35 futures options have a strong predictive ability for subsequent realized volatility in the underlying futures [7]. Furthermore, Jiang and Tian (2005) validate the predictive capacity of model-free IV for future volatility [16]. Busch et al. (2011), Byun and Kim (2013), and Haugom et al. (2014) demonstrate the predictive power of IV using a heterogeneous autoregressive (HAR) model [8, 9, 17]. Pan et al. (2016) find that incorporating IV can enhance the forecasting accuracy of stock volatility within the GARCH model framework [18].

Transitioning toward term structures, Chang et al. (2016) show that the information gained in VIX term structure significantly aids in predicting the volatility of S&P 500 index returns, whereas Wang and Yen (2017) confirm that the VIX term structure is essential in deciding the S& P 500 index future excess returns [19, 20]. Ornelas and Mauad (2019) provide empirical evidence of the predictive ability of the IV term structure in forecasting exchange rate returns [21]. Asensio (2019) examines the VIX futures term structure and its ability to predict directional changes in the VIX, focusing on the expectations hypothesis and profitability of spread trades [22]. Clements et al. (2022) use a HAR model to affirm that the information derived from the IV term structure possesses an exceptional predictive ability for forecasting both the level and directional changes in stock return volatility, even during the volatile period of the Global Financial crisis [23]. Finally, Chen et al. (2023) employ a CHH model and confirm that the volatility factor constructed from swaptions IV term structures has a significant predictive ability for excess bond returns [24].

### 2.2 Applications of machine learning approaches

Machine learning approaches are being increasingly harnessed to broaden the horizons of research across diverse fields. Korkmaz et al. (2023) assess the predictive performance of various machine-learning models for tool wear in the sustainable machining of Bohler steel [25]. Chauhuan (2024) presents an innovative method for analyzing the friction forces in a Ti-6Al-4V alloy utilizing a support vector machine (SVM) optimized by crayfish and arithmetic optimization algorithms [26]. Vashishtha et al. (2023) detail a novel fault diagnosis approach for worm gearboxes by integrating Convolutional Neural Networks with an amended Gorilla troop optimization [27]. See alsoVashishtha(2022) and Guleria (2022) [28, 29].

Despite the boom in machine-learning algorithms in other fields, relatively few studies apply machine-learning approaches to exploit VIX-related issues. Hosker et al. (2018) find that recurrent neural networks (RNNs) and long short-term memory (LSTM) show the best performance for forecasting 1-month VIX futures 3 and 5 day ahead using a principal components analysis and ARIMA model [30]. Guizzardi and Palladini (2019) employ a neural network model to predict VIX futures open-to-close returns and discover that the neural network model is more profitable than the logistic specification and HAR models [13]. Hirsa et al. (2021) apply neural network models (random forest, support vector machines, feed-forward neural networks, and LSTM) to investigate approaches that could replicate the VIX index and VIX futures with fewer options than the original CBOE methodology [31]. Vrontos (2021) utilizes a variety of machine learning models and finds that these models are statistically and economically more effective than traditional econometric models for forecasting directional changes in the VIX [14]. Finally, Avellaneda et al. (2021) generate VIX futures trading signals by including the functional dependence of VIX term structures, VIX futures position, and expected utility in neural networks and find that this approach shows better portfolio performance in out-of-sample backtesting [15].

## 3 Research methodologies

### 3.1 Trading signals construction

Based on Chicago Board Options Exchange (CBOE) calculation algorithms [2], the price level of VIX is based on the prices of options on the S & P 500 index. Let *t* denote time, and let VIX_*t*_ represent the price level of the VIX on that date. At time *t*, the prices of VIX futures with different maturities are represented by Eq 1:

$$\begin{array}{c} F_{t}^{i}≔\text{VIX}\;\text{futures}\;\text{with}\;T_{i}\;\text{maturity}\;\text{days}\;\text{at}\;\text{time}\;\text{t,} \end{array}$$ (1)

where *i* denotes the index number of VIX futures contracts on the ascending expiration day *T*_1_ < *T*_2_ < … < *T*_*d*_.

The term structures of constant-maturity VIX CMFs, spanning maturities from 1 to 6 months in our case, are derived through a linear interpolation method. Linear interpolation employs the two VIX futures contracts closest to the expiration date to construct the term structure, as in Eq 2:

$$\begin{array}{c} V_{t}^{j}≔\omega_{t}^{j}F_{t}^{L_{j}}\;+\left(1-\omega_{t}^{j}\right)F_{t}^{R_{j}}\text{;}\;\text{for}\;\;\text{j}\;\;\text{in}\;\{1,2,\ldots ,6\}, \end{array}$$ (2)

where *L*_*j*_ represents VIX futures with the nearest expiration dates close to *j*-month, but expires sooner than *j*-month. *R*_*j*_ represents VIX futures with the nearest expiration dates close to *j*-month but that expire later than *j*-month, and $\omega_{t}^{j}=\frac{T_{R_{j}}-30j}{T_{R_{j}}-T_{L_{j}}}$. Note that *VIX*_*t*_ is similar to a zero-horizon CMF and CMFs commonly do not suffer fluctuations caused by contract expiry.

#### 3.1.1 Rolling VIX futures derivatives and strategies

By using rolling VIX futures derivatives or strategies, we can replicate the daily returns of CMFs. A quick remark is that SPVXSP, SPVIX2ME, SPVIX3ME, SPVIX4ME, SPVXMP, and SPVIX6ME represent 1–6 month CMFs, respectively. These can be traded either with published derivatives such as ETFs or ETNs or replicated with rolling VIX futures strategies. Specifically, these rolling strategies adjust the CMF weights in Eq 2 to achieve fixed maturity *j*−month. For each *j*, we denote *R*^*j*^ as the daily return on the rolling VIX futures strategy, as in Eq 3, without trading cost:

$$\begin{array}{c} R_{t}^{j}=\frac{\omega_{t}^{j}\Delta F_{t}^{L_{j}}\;+\left(1-\omega_{t}^{j}\right)\Delta F_{t}^{R_{j}}}{\omega_{t}^{j}F_{t}^{L_{j}}\;+\left(1-\omega_{t}^{j}\right)F_{t}^{R_{j}}}, \end{array}$$ (3)

where $\Delta F_{t}^{L_{j}}=F_{t+1}^{L_{j}}-F_{t}^{L_{j}}$. We denote $\Delta t=\frac{1}{252}$, Eq 4 indicates the transformed equation in terms of CMFs:

$${\text{R}}_{\text{t}}^{\text{j}}=\frac{\Delta V_{t}^{j}}{V_{t}^{j}}-\frac{F_{t+1}^{R_{j}}-F_{t+1}^{L_{j}}}{V_{t}^{j}(T_{R_{j}}-T_{L_{j}})\Delta t}\Delta t.$$ (4)

Let Roll denote the drift term in Eq 4, which is the roll yield of the rolling VIX futures strategy:

$$\begin{array}{c} {\text{Roll}}_{t+1}^{j}≔-\frac{F_{t+1}^{R_{j}}-F_{t+1}^{L_{j}}}{V_{t}^{j}\left(T_{R_{j}}-T_{L_{j}}\right)\Delta t}. \end{array}$$ (5)

In contango circumstances, $F_{t+1}^{R_{j}}>F_{t+1}^{L_{j}}$ means *Roll* < 0. Conversely, in backwardation circumstances, $F_{t+1}^{R_{j}}<F_{t+1}^{L_{j}}$ implies that *Roll* > 0. We re-write Eq 5 as follows:

$${\text{R}}_{\text{t}}^{\text{j}}=\frac{\Delta V_{t}^{j}}{V_{t}^{j}}+Roll_{t}^{j}\Delta t.$$ (6)

From Eq 6, we see $R_{t}^{j}$ consists of two parts: the change in $V_{t}^{j}$ and roll yield. As previously discussed, the most likely VIX futures term structures are in contango, which explains why the value of rolling VIX futures strategies decays.

Given the mean-reversion property of volatility, ideally, suppose $V_{t}^{j}$ and CMFs are both stable for long-period; then, the return on $V_{t}^{j}$ should be $V_{t}^{j}-V_{t}^{j-1}$. Thus, we denote *μ* as Eq 7, and $\mu_{t}^{j}$ contains instantaneous market views of return about $V_{t}^{j}$.:

$$\begin{array}{c} \mu_{t}^{j}=V_{t}^{j}-V_{t}^{j-1}. \end{array}$$ (7)

### 3.2 Proposed machine learning algorithms

#### 3.2.1 XGBoost

The concept of boosting proposed by Freund and Schapire (1999) involves an ensemble model strategy that combines multiple weak learners to create more robust learners [32]. Friedman (2001) introduces Gradient Tree Boosting, an improvement in boosting that incorporates a gradient descent algorithm into the loss function to minimize errors [33]. Expanding on gradient boosting, Chen and Guestrin (2016) introduce XGBoost, a scalable end-to-end tree-boosting system based on an additive training strategy that augments gradient tree boosting with regularization, efficient handling of sparse data, and enhanced computational efficiency, resulting in superior speed and accuracy [34]. Eq 9 denotes the objective function of XGBoost, where $\sum_{i=1}^{n}l(y_{i},{\hat{y}}_{i})$ represents the training loss function and $\sum_{k=1}^{K}\Omega (f_{k})$ denotes the complexity of trees.

$$\begin{array}{c} \text{Obj}=\underset{Training\;Loss}{\underset{︸}{\sum_{i=1}^{n}l\left(y_{i},{\hat{y}}_{i}\right)}}+\underset{Complexity\;of\;trees}{\underset{︸}{\sum_{k=1}^{K}\Omega \left(f_{k}\right)}} \end{array}$$ (8)

#### 3.2.2 Multilayer perceptrons

The Multi-Layer Perceptron (MLP) includes at least three layers of nodes: an input layer, a hidden layer, and an output layer. Unlike a linear perceptron, each node (or neuron) in an MLP uses a nonlinear activation function. This aspect of MLP allows it to distinguish data that are not linearly separable, which is a limitation of the basic perceptron. MLPs are widely used to solve complex problems in machine learning by learning nonlinear function approximator for classification or regression. They are trained using back propagation, ensuring that the error between the actual and predicted outputs is minimized. A detailed explanation of MLP can be found in Haykin’s (2001) book [35]. In our experiment, we use the leaky ReLU introduced by Maas et al. (2013) as the activation function *ϕ* for MLP, which we show mathematically in Eq 9 [36]. Given the activation function *ϕ* and features *X* ∈ *R*^*n*×*d*^, where *x* represents the batch size and *d* represents the dimensionalities,

$$\begin{array}{c} \phi^{\left(i\right)}=\text{max}\left({W^{\left(i\right)}}^{T}x,0\right)=\{\begin{array}{cc} {W^{\left(i\right)}}^{T}x,\; & {W^{\left(i\right)}}^{T}x>0\\ 0,\;else, \end{array} \end{array}$$ (9)

where *W*^(*i*)^ is the weight vector of the *i*_*th*_ hidden unit and *x* is the input feature.

#### 3.2.3 Recurrent Neural Network (RNN)

Multiple machine learning approaches have also been introduced based on RNN architecture. RNN aims to find patterns in sequences of data. Schmidt (2019) provides a comprehensive overview of the basic architecture and functions of RNNs whereas Sherstinsky (2023) focuses on deriving a classical RNN formulation from differential equations and addresses the challenges in training standard RNNs [37, 38]. The transformation of RNN into a “Vanilla LSTM” network is explained through logicial arguments with a detailed description of the LSTM system’s equations and entities. The RNN training function *f* is

$$\begin{array}{c} h_{t},y_{t}=f\left(h_{t-1},x_{t}\right), \end{array}$$ (10)

where *h* represents the hidden layer, which carries sequence information by *t* − 1, *x*_*t*_ is the input at *t*, *f* outputs the result *y*_*t*_ at *t* and the sequence information *h*_*t*_.

#### 3.2.4 Long Short-Term Memory (LSTM)

The LSTM model originally proposed by Hochreiter and Schmidhuber (1997) represents a significant milestone in the field of neural networks, particularly for handling sequential data [39]. LSTMs were designed to overcome the limitations of traditional RNNs, particularly those related to learning long-range dependencies. Traditional RNNs face vanishing and exploding gradient problems, making it challenging to retain information over long sequences. The unique memory cells and input, forget, and output gate mechanisms enable LSTM to store important information and forget irrelevant details over long sequences. With recent innovations in attention mechanisms, Wang and Hao (2020) introduce the attention-based LSTM (ALSTM) model, which integrates multi-head dot product attention within the LSTM architecture and significantly enhances the model’s ability for complex reasoning over sequences [40].

Eqs 12, 13 and 14 illustrate the mathematical details for the implementation of LSTM and ALSTM in our study. The LSTM framework denotes *c* as the long-term sequence information that distinguishes it from *h* and designs gates to filter information. A gate cell can be represented as

$$\begin{array}{c} g=\sigma (W•\left[h_{t-1},x_{t}\right]+b), \end{array}$$ (11)

where *W*, *b* are trainable parameters, *σ* represents the activation function, and *g* can be used as a gate. The LSTM framework has the following three gates on RNN:

$$\begin{array}{c} \left\{ \begin{array}{c} c_{t}=g^{f}⊙c_{t-1}-g^{i}⊙tanh\left(W•\left[h_{t-1},x_{t}\right]+b\right)\\ h^{t}=g^{o}⊙tanh\left(c_{t}\right)\\ y^{t}=f\left(h_{t}\right) \end{array}\right., \end{array}$$ (12)

where *g*^*f*^, *g*^*i*^, *andg*^*o*^ represent the three gates and *f* is a function that links LSTM outputs to subsequent tasks.

The ALSTM adds encoder and decoder attention-based layers to the LSTM. Given *n* series, under one timestamp, the stage one encoder layer enables attention mechanisms and transformation to new input simultaneously.

$$\begin{array}{c} \left\{ \begin{array}{c} e_{t}^{k}=tanh\left(W•\left[h_{t-1},c_{t-1}\right]+bx^{k}\right)\\ \alpha_{t}^{k}=\frac{exp(e_{t}^{k})}{\sum_{i=1}^{n}exp\left(e_{t}^{i}\right)}\\ \tilde{x_{t}}=\left(\alpha_{t}^{1}x_{t}^{1},\alpha_{t}^{2}x_{t}^{2},\ldots ,\alpha_{t}^{n}x_{t}^{n}\right), \end{array}\right. \end{array}$$ (13)

with $\tilde{x_{t}}$ as the input, the first stage constructs an LSTM framework called *L*_1_, and the second stage decoder layer learns attention with all hidden states *h*_*t*_ of *L*_1_. The output $\tilde{h_{t}}$ transforms the label *y* of another LSTM framework, namely *L*_2_, such that the two attention layers can learn the relations between *n* series. Let *d* denote the hidden state of *L*_2_; then, the transforms of *y* can be formulated as

$$\begin{array}{c} \left\{ \begin{array}{c} l_{t}=tanh\left(W•\left[d_{t-1},c_{t-1}^{2}\right]+bh^{i}\right)\\ \beta_{t}=\frac{exp(l_{t})}{\sum_{i=1}^{T}exp\left(\beta_{t}\right)}\\ c_{t}=\sum_{i=1}^{T}\beta_{i}h_{i}\\ \tilde{y_{t-1}}=W•\left[y_{t-1};c_{t-1}\right]+b \end{array}\right., \end{array}$$ (14)

where $\tilde{y}$ is the input to *L*_2_.

#### 3.2.5 Gated Recurrent Unit

The Gated Recurrent Unit (GRU), first introduced by Gulcehre et al. (2014), is designed to efficiently capture temporal dependencies in sequential data [41]. The efficiency of the GRU is achieved through its unique architecture, which consists of two gates: an update gate and a reset gate. The update gate plays a crucial role in determining how much of its previous state the GRU retains, thereby enabling the effective capture of long-term dependencies. Conversely, the reset gate influences the amount of past information that is forgotten, assisting the model in concentrating on the most pertinent information. These gates effectively control the flow of information within the unit, allowing the network to retain important information from past data inputs while discarding irrelevant data. This selective memory mechanism empowers the GRU to effectively tackle the vanishing gradient problem, a common challenge in traditional RNNs. The implementation details can be found in Eq 15.

$$\begin{array}{c} \left\{ \begin{array}{c} h_{t-1}^{\prime }=h_{t-1}•g^{r}\\ h^{\prime }=tanh\left(W•\left[h_{t-1}^{\prime },x_{t}\right]+b\right)\\ h^{t}=\left(1-g^{z}\right)⊙tanh\left(c_{t}\right)+g^{z}⊙h^{\prime } \end{array}\right. \end{array}$$ (15)

Following the notation used in LSTM, *g* represents the gate cell denoted in Eq 11.

#### 3.2.6 LightGBM

LightGBM is an algorithmic framework for gradient-boosting decision trees (GBDT) proposed by Ke et al. (2017) [42]. It supports efficient parallel computation and offers advantages such as faster training speed and lower memory consumption. This primarily incorporates two algorithmic improvements: gradient-based one-sided sampling (GOSS) and exclusive feature bundling (EFB). GOSS retains instances with large gradients and performs random sampling on those with small gradients. It then adjusts the new sampling with a weight adjustment to ensure that the information gain remains unchanged. This approach allows for the calculation of information gain using only the remaining data with high gradients. Compared with XGBoost, which traverses all feature values, Algorithm 1 saves a considerable amount of time and space.

**Algorithm 1** Gradient-based One-Side Sampling

**Input:** *I*: training data, *d*: iterations

**Input:** *a*: sampling ratio of large gradient data

**Input:** *b*: sampling ratio of small gradient data

**Input:** *loss*: loss function, *L*: weak learner

 $models\leftarrow ,fact\leftarrow \frac{1-a}{b}$

 *topN* ← *a* × *len*(*I*), *randN* ← *b* × *len*(*I*)

 **for** *i* = 1 → *d* **do**

  *preds* ← *models*.*predict*(*I*)

  *g* ← *loss*(*I*, *preds*), *ω* ← *EqualWeight*

  *sorted* ← *GetSortedIndices*(*abs*(*g*))

  *topSet* ← *sorted*[1: *topN*]

  *randSet* ← *RandomPick*(*sorted*[*topN*: *len*(*I*)], *randN*)

  *usedSet* ← *topSet*+ *randSet*

  *ω*[*randSet*]× = *fact*

  *newModel* ← *L*(*I*[*usedSet*], *g*[*usedSet*], *ω*[*usedSet*])

  *models*.*append*(*newModel*)

 **end for**

High-dimensional data are typically mutually exclusive. EFB can safely bundle exclusive features into a single feature using the designed feature-scanning algorithm 2.

**Algorithm 2** Merge Exclusive Features

**Input:** *numData*: number of data

**Input:** *F*: One bundle of exclusive features

 *binRanges* ← 0, *totalBin* ← 0

 **for** *f* ← *F* **do**

  *totalBin*+ = *f*.*numBin*

  *binRanges*.*append*(*totalBin*)

 **end for**

 **for** *i* = 1 → *numData* **do**

  *newBin*[*i*] ← 0

  **for** *j* = 1 → *len*(*F*) **do**

   **if** *F*[*j*].*bin*[*i*] ≠ 0 **then**

    *newBin*[*i*] ← *F*[*j*].*bin*[*i*] + *binRanges*[*j*]

   **end if**

  **end for**

 **end for**

**Output:** newBing, binRanges

## 4 Data preparation and experiment design

### 4.1. Data preparation

In line with our primary goal of predicting the next day returns of CMFs. it is rational to incorporate the CMFs-related features $V_{t}^{j}$ and $Roll_{t}^{j}$ as detailed in Eqs 2 and 5 Additionally, to thoroughly examine the predictive ability of term structures, we introduce $\mu_{t}^{j}$ and $△Roll_{t}^{j}$ to provides a comprehensive description of the CMFs term structure. We also integrate *TLT* and *SPY* into our analysis to partially represent the macroeconomic conditions. Furthermore, various statistically derived features were employed to explore possible additional information, with the details provided in Table 5. The sample period is December 20, 2005 to August 7, 2022. Three distinct feature sets are utilized to incrementally examine the predictive ability of VIX term structures and the additional information offered by statistically derived features. The detailed descriptions of the three feature sets are presented in Tables 3–5.

**Table 3:** Simple feature sets.

VIX-CMFs	CMFs-related features	Macro-features
*CMF*_1_	$[V_{t}^{1},Roll_{t}^{1}]$	$\left[ln_{SPY_{t}},ln_{TLT_{t}},VIX_{t}\right]$
*CMF*_2_	$[V_{t}^{2},Roll_{t}^{2}]$	$\left[ln_{SPY_{t}},ln_{TLT_{t}},VIX_{t}\right]$
*CMF*_3_	$[V_{t}^{3},Roll_{t}^{3}]$	$\left[ln_{SPY_{t}},ln_{TLT_{t}},VIX_{t}\right]$
*CMF*_4_	$[V_{t}^{4},Roll_{t}^{4}]$	$\left[ln_{SPY_{t}},ln_{TLT_{t}},VIX_{t}\right]$
*CMF*_5_	$[V_{t}^{5},Roll_{t}^{5}]$	$\left[ln_{SPY_{t}},ln_{TLT_{t}},VIX_{t}\right]$
*CMF*_6_	$[V_{t}^{6},Roll_{t}^{6}]$	$\left[ln_{SPY_{t}},ln_{TLT_{t}},VIX_{t}\right]$

*CMF*_*j*_ represents CMFs with *j* months time to expiry.

**Table 4:** Term structure feature sets.

*VIX*_*CMFs*_	CMFs-related features	Macro-features	Term Structures features
*CMF*_1_	$[V_{t}^{1},Roll_{t}^{1}]$	$\left[ln_{SPY_{t}},ln_{TLT_{t}},VIX_{t}\right]$	$\left[\mu_{t}^{1},△Roll_{t}^{1}\right]$
*CMF*_2_	$[V_{t}^{2},Roll_{t}^{2}]$	$\left[ln_{SPY_{t}},ln_{TLT_{t}},VIX_{t}\right]$	$\left[\mu_{t}^{2},△Roll_{t}^{2}\right]$
*CMF*_3_	$[V_{t}^{3},Roll_{t}^{3}]$	$\left[ln_{SPY_{t}},ln_{TLT_{t}},VIX_{t}\right]$	$\left[\mu_{t}^{3},△Roll_{t}^{3}\right]$
*CMF*_4_	$[V_{t}^{4},Roll_{t}^{4}]$	$\left[ln_{SPY_{t}},ln_{TLT_{t}},VIX_{t}\right]$	$\left[\mu_{t}^{4},△Roll_{t}^{4}\right]$
*CMF*_5_	$[V_{t}^{5},Roll_{t}^{5}]$	$\left[ln_{SPY_{t}},ln_{TLT_{t}},VIX_{t}\right]$	$\left[\mu_{t}^{5},△Roll_{t}^{5}\right]$
*CMF*_6_	$[V_{t}^{6},Roll_{t}^{6}]$	$\left[ln_{SPY_{t}},ln_{TLT_{t}},VIX_{t}\right]$	$\left[\mu_{t}^{6},△Roll_{t}^{6}\right]$

**Table 5:** Statistically derived feature sets.

*VIX*_*CMFs*_	CMFs-related features	Macro-features	Term Structure features	statistical-derived features
*CMF*_1_	$[V_{t}^{1},Roll_{t}^{1}]$	$\left[ln_{SPY_{t}},ln_{TLT_{t}},VIX_{t}\right]$	$\left[\mu_{t}^{1},△Roll_{t}^{1}\right]$	$\left[Std_{T}(All_{t}^{1}),Skew_{T}^{1}(All_{t}^{1}),Kurt_{T}(All_{t}^{1}),Mean_{T}(All_{t}^{1}\right)$ , $z\;score_{T}(term_{t}^{1})$ , *where T* ∈ {5, 20, 60}]
*CMF*_2_	$[V_{t}^{2},Roll_{t}^{2}]$	$\left[ln_{SPY_{t}},ln_{TLT_{t}},VIX_{t}\right]$	$\left[\mu_{t}^{2},△Roll_{t}^{2}\right]$	$\left[Std_{T}(All_{t}^{2}),Skew_{T}^{2}(All_{t}^{2}),Kurt_{T}(All_{t}^{2}),Mean_{T}(All_{t}^{2}\right)$ , $z\;score_{T}(term_{t}^{2})$ , *where T* ∈ {5, 20, 60}]
*CMF*_3_	$[V_{t}^{3},Roll_{t}^{3}]$	$\left[ln_{SPY_{t}},ln_{TLT_{t}},VIX_{t}\right]$	$\left[\mu_{t}^{3},△Roll_{t}^{3}\right]$	$\left[Std_{T}(All_{t}^{3}),Skew_{T}^{3}(All_{t}^{3}),Kurt_{T}(All_{t}^{3}),Mean_{T}(All_{t}^{3}\right)$ , $z\;score_{T}(term_{t}^{3})$ , *where T* ∈ {5, 20, 60}]
*CMF*_4_	$[V_{t}^{4},Roll_{t}^{4}]$	$\left[ln_{SPY_{t}},ln_{TLT_{t}},VIX_{t}\right]$	$\left[\mu_{t}^{4},△Roll_{t}^{4}\right]$	$\left[Std_{T}(All_{t}^{4}),Skew_{T}^{4}(All_{t}^{4}),Kurt_{T}(All_{t}^{4}),Mean_{T}(All_{t}^{4}\right)$ , $z\;score_{T}(term_{t}^{4})$ , *where T* ∈ {5, 20, 60}]
*CMF*_5_	$[V_{t}^{5},Roll_{t}^{5}]$	$\left[ln_{SPY_{t}},ln_{TLT_{t}},VIX_{t}\right]$	$\left[\mu_{t}^{5},△Roll_{t}^{5}\right]$	$\left[Std_{T}(All_{t}^{5}),Skew_{T}^{5}(All_{t}^{5}),Kurt_{T}(All_{t}^{5}),Mean_{T}(All_{t}^{5}\right)$ , $z\;score_{T}(term_{t}^{5})$ , *where T* ∈ {5, 20, 60}]
*CMF*_6_	$[V_{t}^{6},Roll_{t}^{6}]$	$\left[ln_{SPY_{t}},ln_{TLT_{t}},VIX_{t}\right]$	$\left[\mu_{t}^{6},△Roll_{t}^{6}\right]$	$\left[Std_{T}(All_{t}^{6}),Skew_{T}^{6}(All_{t}^{6}),Kurt_{T}(All_{t}^{6}),Mean_{T}(All_{t}^{6}\right)$ , $z\;score_{T}(term_{t}^{6})$ , *where T* ∈ {5, 20, 60}]

$All_{t}^{j}\in \{CMFs\;related\;features,Macro\;features,Term\;Structure\;features\}$ and $term_{t}^{j}\in \{Term\;Structure\;features\}$

### 4.2. Empirical experiment design

The primary aim of this empirical experiment was to assess the predictive ability and backtesting performance of machine learning models, specifically focusing on the prediction and backtesting enhancements afforded by the incorporation of term structure and statistically derived features. We also aim to evaluate the backtesting performance of the C-MVO and long-short rank-based strategy.

Prior to machine learning model training phases, our approach involves processing data from six distinct CMFs at each timestamp, which include both the prediction labels and features in Tables 3–5. Drawing on Prado’s (2018) concept of “feature stacking,” we combine 6 series of data (*x*_*i*_, *y*_*i*_)_{*i* = 1, …,6}_ into a singular dataset (*X*, *y*) [43]. This strategy is instrumental in diminishing the model bias, particularly that associated with outliers and overfitting. During the machine-learning model training phase, cross-validation (CV) has been extensively employed to determine the generalization error of models in other studies. However, when applied to time-series data, CV faces significant challenges due to the potential for future information leakage. This is primarily due to the inclusion of future data points via random sampling, along with the inherent temporal dependencies found in financial time-series data. Therefore, to avoid future information leakage, we adopt the walk-forward method proposed by Prado (2018) and implement a walk-forward training and backtesting procedure, as detailed in algorithms 3 and 4 [43].

In this experiment, the dataset is divided as follows. The training dataset encompass the period from December 20, 2005, to June 30, 2010. The validation dataset covers the dates from July 1, 2010, to December 31, 2010, and the test dataset extends from January 1, 2011, to August 15, 2022. The rationale for this data segmentation is as follows.

1. Ensure an extensive backtesting period of 11 years
2. Guaranteeing a substantial initial training dataset and the inclusion of extreme market scenarios such as the 2008 global financial crisis.
3. Establish a six-month validation set to confirm that the training data for predictions are at least six months old, thereby averting information leakage and bolstering robustness. Employing more recent data may enhance the results.
4. Following an expanding time-window process when using the data, meaning that each training session encompasses all preceding data points, thus fostering a more robust predictive model.

**Algorithm 3** Walk-Forward Procedure

*rollingtasks* ← *Algorithm TaskRollGen*4

**for** *task* ← *rollingtasks* **do**

 $model\overset{fit}{\leftarrow }task[train]$

 $pred\overset{predict}{\leftarrow }task[test]$

 $preds\overset{append}{\leftarrow }pred$

**end for**

$PRED\overset{concat}{\leftarrow }preds$ # PRED time horizon is equal to Task[test]’s time horizons

$metrics\overset{calculate}{\leftarrow }PRED$

*backtestingresult* ← *PRED, strategy # bar by bar*

**Algorithm 4** Task Roll Gen

**Input:** TASK contains {TRAIN, VALID, TEST, roll_length}

**Output:** rolling tasks with their own {train, valid, test}

*fragments* ← Test divided by roll_length

**for** *m* ← *fragments* **do**

 *task* ← *Task*

 *task*[*test*]←*m*

 *task*[*valid*]←[*start*_*date*_*test*_ − 1 −*lenth*_*Valid*_, *start*_*date*_*test*_ − 1]

 *task*[*train*]←[*start*_*date*_*TRAIN*_, *strat*_*date*_*valid*_ − 1]   # expanding

 $rollingtasks\overset{append}{\leftarrow }task$

**end for**

### 4.3. C-MVO trading strategy & long-short trading strategy

To evaluate trading performance using numerical predictions from all machine learning models, we introduce our newly proposed C-MVO and long-short trading strategies. Detailed explanations of these strategies are provided below.

The C-MVO strategy aligns with the classical mean-variance portfolio optimization framework initially proposed by Markowitz (1952) [44]. For the C-MVO strategy, we incorporate several additional constraints. *R*_*i*_ represents the numerical predictions of six VIX CMFs next-day returns. *Cov*_*port*_ is derived using a 60 days historical covariance matrix. The risk-aversion parameter, denoted as *γ*, is fixed at 0.2. To ensure effective risk management, we impose constraints based on portfolio characteristics. The C-MVO strategy limits gross leverage to a maximum of three and enforces absolute caps on the individual weights of CMFs, ensuring that no individual weight exceeds the absolute value of 1. Furthermore, a critical risk threshold is established, we constrain the portfolio’s annualized risk to not exceed 30%. This strategy provides a rigorous approach for portfolio construction, striking a balance between expected returns and risk mitigation. The application of historical covariance data and delineation of precise risk parameters contribute to the robustness and reliability of the derived portfolio weights, thus enhancing the integrity of the investment strategy. The detailed optimization function is given by Eq 16. The long-short strategy, a benchmark rank-based trading strategy, aims to exploit profits from the relative performance of assets through a combination of long and short positions. In our trading strategy design, we first rank six VIX CMFs instruments based on predicted returns in descending order; we only consider VIX CMFs with the highest and lowest predicted returns. On each trading day, we allocate a 50% long position to the highest-predicted-returns VIX CMF and 50% short position to the lowest-predicted-returns VIX CMF.

$$\begin{array}{cc} \underset{w_{i}}{\text{max}}:\; & \sum_{i=1}^{n}w_{i}R_{i}-\gamma \times \text{risk}\\ \text{s.t.:}\; & \underset{i}{\text{max}}\left|w_{i}\right|\leq 1\\ & \sum_{i=1}^{n}\left|w_{i}\right|\leq 3\\ & |\sum_{i=1}^{n}w_{i}|\leq 2\\ & \text{risk}\leq {\text{risk}}_{\text{max}}\\ & \text{where}\;\text{risk}=w^{T}{\text{Cov}}_{\text{port}}w,\\ & {\text{risk}}_{\text{max}}=0.3,\;\gamma =0.2 \end{array}$$ (16)

## 5 Results & discussion

### 5.1. Evaluation metrics

Given that the experiment aims to evaluate machine learning models’ predictive ability and subsequent trading strategy profitability, we first employ a variety of metrics to evaluate the predictive performance in the realm of quantitative trading. The primary metric utilized is the information coefficient (IC), a statistical measure derived from the Pearson correlation coefficient, which quantifies the correlation between predicted returns $\hat{y}$ and subsequent realized returns *y* of the portfolio, denoted as $\text{Corr}(\hat{y},y)$. A high positive IC indicates that the model predictions are highly correlated with actual returns, suggesting a strong predictive ability. Conversely, a low or negative IC suggests inaccuracies in the prediction, or even contrarian of actual outcomes. A detailed discussion of IC can be found in Goodwin’s (1998) work [45]. In addition, we incorporate rank IC, which calculates the Pearson correlation coefficient using predicted and actual ranks rather than actual returns, represented as $\text{Corr}(\hat{ranks},ranks)$. Moreover, we integrate IC information ratio (ICIR) into our evaluation that illustrates the stability of models predictive ability, ICIR can be calculated as $ICIR\approx \frac{\bar{IC}}{\text{Std}(IC)}$, with a similar formulation for rank ICIR, replacing IC with rank IC, denoted as $\text{Rank}\;\text{ICIR}\approx \frac{\bar{Rank\;IC}}{\text{Std}(Rank\;IC)}$. To assess the backtesting results of the C-MVO strategy and long-short strategy, the information ratio is denoted as $\text{IR}=\frac{{\text{port}}_{\text{return}}}{{\text{std}}_{\text{return}}}$ and annualized returns are included in the metrics.

### 5.2. VIX CMFs term structure predictive ability evaluation

This section focuses on evaluating the predictive ability of the CMFs’ term structure features Δ*roll* and *μ* and other statistical features listed in Table 5. Table 6 lists the combined results of the predictive and backtesting performance of the three feature sets.

**Table 6:** Results summary.

model	feature sets	IC	ICIR	Rank IC	Rank ICIR	C-MVO Annualized Returns	C-MVO Information Ratio	Long Short Annualized Returns	Long Short Information Ratio
ALSTM	Statistical-derived	0.040	0.066	0.027	0.048	-0.002	-0.016	0.033	0.174
	Simple	0.005	0.007	-0.005	-0.008	0.057	0.803	0.051	0.258
	TermStructure	0.029	0.042	0.026	0.041	0.071	0.744	0.112	0.579
GRU	Statistical-derived	0.021	0.035	0.017	0.031	0.049	0.310	0.096	0.641
	Simple	-0.023	-0.033	-0.026	-0.040	0.045	0.517	0.059	0.310
	TermStructure	0.004	0.006	0.002	0.004	0.028	0.241	0.080	0.409
LightGBM	Statistical-derived	0.019	0.059	0.085	0.127	0.020	0.264	-0.085	-0.355
	Simple	-0.024	-0.050	-0.044	-0.069	-0.004	-0.044	-0.121	-0.511
	TermStructure	0.035	0.066	0.054	0.079	0.023	0.327	-0.085	-0.367
LSTM	Statistical-derived	0.040	0.067	0.036	0.064	0.047	0.416	0.020	0.123
	Simple	-0.015	-0.022	-0.026	-0.042	0.030	0.388	0.044	0.262
	TermStructure	0.021	0.031	0.016	0.026	0.059	0.671	0.034	0.168
Linear	Statistical-derived	0.095	0.147	0.079	0.140	0.165	1.049	0.117	0.646
	Simple	0.090	0.147	0.070	0.131	0.068	1.325	0.186	0.992
	TermStructure	0.114	0.162	0.091	0.152	0.150	2.291	0.214	1.105
MLP	Statistical-derived	-0.011	-0.018	-0.013	-0.024	-0.023	-0.074	0.056	0.325
	Simple	-0.002	-0.004	-0.001	-0.001	0.195	0.630	-0.009	-0.046
	TermStructure	0.011	0.017	0.012	0.020	0.013	0.043	0.050	0.302
XGB	Statistical-derived	0.008	0.012	0.011	0.019	0.062	0.250	0.024	0.141
	Simple	0.015	0.023	0.003	0.005	-0.019	-0.073	0.042	0.260
	TermStructure	0.047	0.071	0.043	0.069	0.011	0.044	0.116	0.632

#### 5.2.1 Term structure feature set

Based on Table 6, it can be observed that all models using simple feature sets exhibit a lower IC than those using feature sets containing term structure features and statistically derived features. Notably, models displaying negative Information Coefficient (IC) values, including GRU (-0.023), LightGBM (-0.024), LSTM (-0.015), and MLP (-0.002), demonstrate a discrepancy between predicted and actual returns when using a simple dataset. Interestingly, when feature sets that included term structure features were incorporated, all models that initially had negative IC values improved, showing positive IC values. In particular, GRU (0.004), LightGBM (0.035), LSTM (0.021), and MLP (0.011). This outcome initially validated the effectiveness of VIX CMFs term structure features in augmenting the predictive ability of the models for the next-day returns of CMFs.

Regarding the backtesting results, for the long-short strategy, most models comprising term structure features showed better information ratios, except LSTM model information ratio decreased from 0.262 to 0.168 by switching feature sets from simple to term structure feature sets. For the C-MVO strategy, the results were slightly unstable; however, most models showed better backtesting results. Based on the foregoing analysis, it can be initially said that term structure features possess a robust ability to predict the next-day returns of VIX CMFs and can enhance the profitability of both trading strategies.

#### 5.2.2 Statistically derived features set

Here, we delve deeper into studying the incremental effects of statistically derived features compared to the original term structure features. Table 7 lists the optimal feature sets for all models. The results suggest that, although increments from statistically derived features do exist, they are relatively minor in magnitude. Therefore, the subsequent discussion focuses solely on the term structure feature set.

**Table 7:** Feature set performance.

model	IC	Rank IC	C-MVO Information Ratio	Long Short Information Ratio
ALSTM	Statistical-derived	Statistical-derived	Simple	TermStructure
GRU	Statistical-derived	Statistical-derived	Simple	Statistical-derived
LGB	TermStructure	Statistical-derived	TermStructure	Statistical-derived
LSTM	Statistical-derived	Statistical-derived	TermStructure	Simple
Linear	TermStructure	TermStructure	TermStructure	TermStructure
MLP	TermStructure	TermStructure	Simple	Statistical-derived
XGB	TermStructure	TermStructure	Statistical-derived	TermStructure

**Fig 1:**
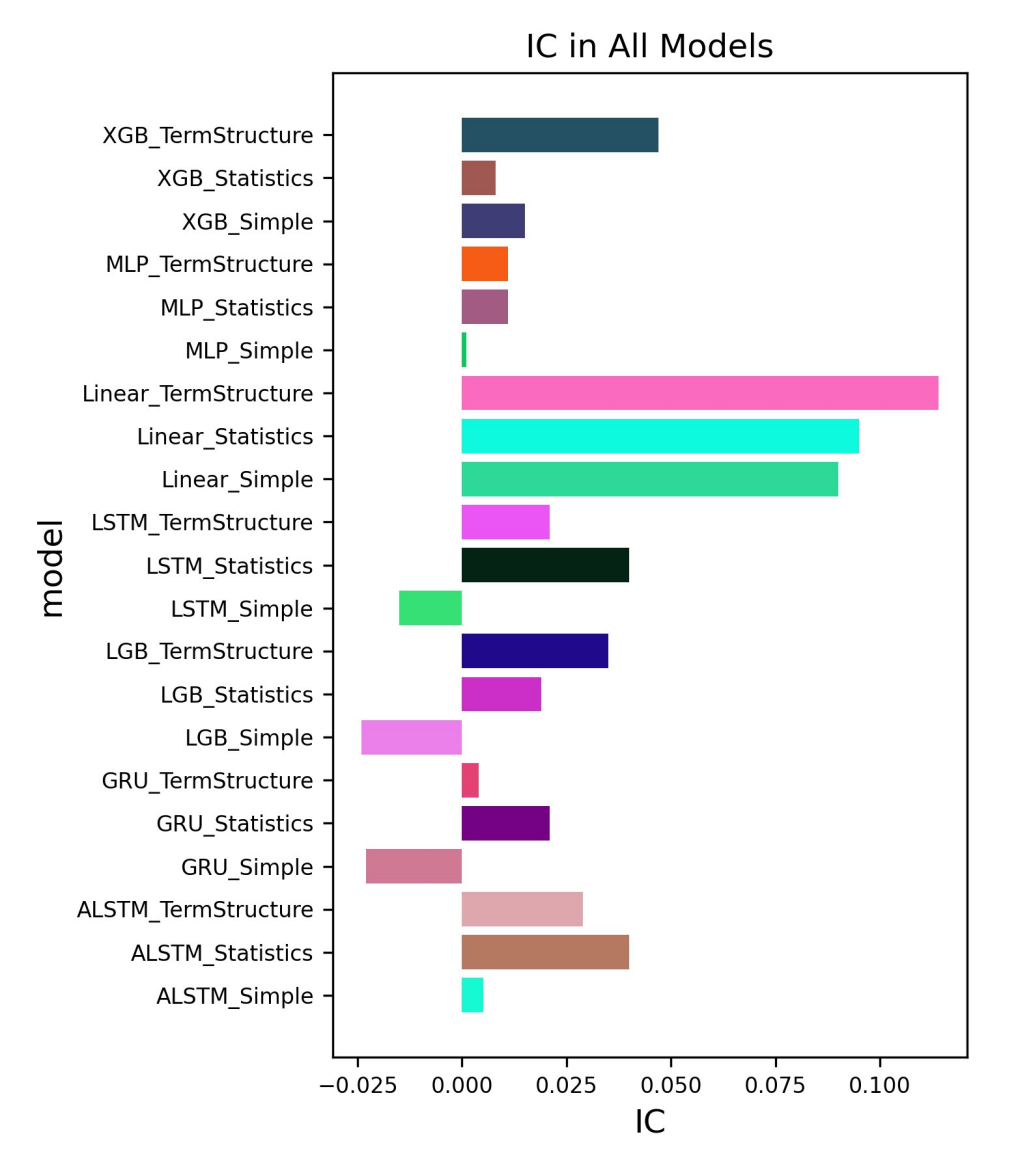
IC in all models.

Given previous findings of VIX CMFs term structure features, the average IC value of 0.037 in Table 8 and the average information ratio of 0.623 in Table 9 further underscore that term structure features can not only facilitate valid VIX CMFs next-day return numerical predictions, but also translate these predicted returns into actionable and profitable trading strategies. Specifically, in our experiment using a linear regression model, term structure features such as Δ*roll* and *μ* directly elevate the IC from 0.09 to 0.114, and the long-short information ratio from 0.992 to 1.105, indicating a significant improvement in performance. Fig 1 shows a bar chart displaying the IC (Information Coefficient) results for all models.

**Table 8:** Predictive performance.

model	IC	ICIR	Rank IC	Rank ICIR
ALSTM	0.029	0.042	0.026	0.041
GRU	0.004	0.006	0.002	0.004
LGB	0.035	0.066	0.054	0.079
LSTM	0.021	0.031	0.016	0.026
Linear	0.114	0.162	0.091	0.152
MLP	0.011	0.017	0.012	0.020
XGB	0.047	0.071	0.043	0.069
mean	0.037	0.056	0.035	0.056

Term structure feature sets only

**Table 9:** Backtesting performance.

model	C-MVO Annualized Return	C-MVO Information Ratio	Long Short Annualized Return	Long Short Information Ratio
ALSTM	0.071	0.744	0.112	0.579
GRU	0.028	0.241	0.080	0.409
LGB	0.023	0.327	-0.085	-0.367
LSTM	0.059	0.671	0.034	0.168
Linear	0.150	2.291	0.214	1.105
MLP	0.013	0.043	0.050	0.302
XGB	0.011	0.044	0.116	0.632
mean	0.051	0.623	0.074	0.404

Term structure feature sets only

### 5.3. Trading strategies evaluation

**Fig 2:**
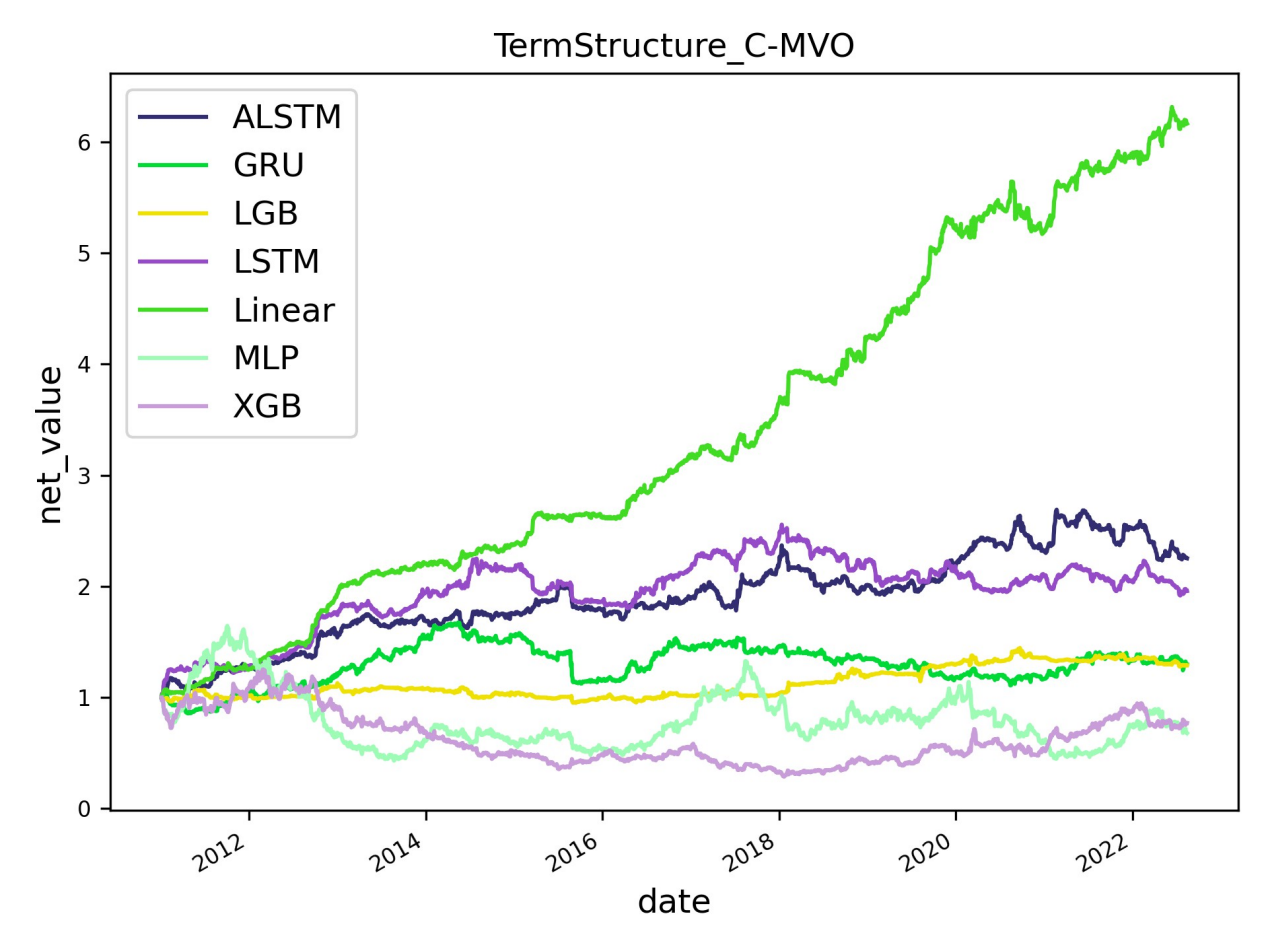
Models’ net values with term structure feature sets using the C-MVO strategy.

Following the established effectiveness of term structure features, this section investigates their impact on trading strategies, specifically focusing on the potential profitability boost offered by our innovative C-MVO trading strategy. Table 9 presents backtesting results for the C-MVO strategy and the benchmark long-short strategy. For individual machine learning models, the information ratios for ALSTM (0.744), LightGBM (0.327), LSTM (0.671), and linear regression (2.291) models surpassed the corresponding performance under the long-short strategy. Meanwhile, the C-MVO strategy’s average information ratio of 0.623, in contrast to the long-short strategy’s average ratio of 0.404, indicates that the C-MVO strategy could bring overall improvements over the latter. Fig 2 shows the net value curves of different models on the term structure features and C-MVO strategy. One plausible explanation for these improvements is that the C-MVO strategy more effectively utilizes the numerical predicted returns of all six CMFs. By contrast, the long-short strategy, which relies solely on ranks derived from these numerical predictions, potentially misses critical information, such as the precise magnitude relationships among the CMFs’ predicted returns. Therefore, the C-MVO strategy could be considered superior to the benchmark rank-based long-short strategy and offers an actionable path for researchers and practitioners to discover the highest possible economic benefits derived from term structure features.

### 5.4. Machine learning model evaluation

This section aims to evaluate the results from the perspective of the machine learning models. To better compare various machine learning models, we classify the models into three categories: RNN-based (GRU, LSTM, ALSTM), tree-based (XGBoost and LightGBM), and vanilla models (Linear Regression, MLP). Tables 10 and 11 list the performance of each model type.

**Table 10:** Models predictive performances.

model-types	models	IC	ICIR	Rank IC	Rank ICIR
Neural-based	ALSTM	0.029	0.042	0.026	0.041
	GRU	0.004	0.006	0.002	0.004
	LSTM	0.021	0.031	0.016	0.026
Tree-based	LightGBM	0.035	0.066	0.054	0.079
	XGBoost	0.047	0.071	0.043	0.069
Vanilla	Linear Regression	0.114	0.162	0.091	0.152
	MLP	0.011	0.017	0.012	0.020

Term Structure feature sets only & group by model types.

**Table 11:** Models backtesting performances.

model_type	model	C-MVO Annualized Return	C-MVO Information Ratio	Long Short Annualized Return	Long Short Information Ratio
RNN_based	ALSTM	0.071	0.744	0.112	0.579
	GRU	0.028	0.241	0.080	0.409
	LSTM	0.059	0.671	0.034	0.168
Tree_based	LightGBM	0.023	0.327	-0.085	-0.367
	XGBoost	0.011	0.044	0.116	0.632
Vanilla	Linear Regression	0.150	2.291	0.214	1.105
	MLP	0.013	0.043	0.050	0.302

Term structure feature sets only & group by models types.

#### 5.4.1 Neural-network-based models

For neural-network-based models, we initially focused on comparing the performance of LSTM and its variant ALSTM. The IC value of LSTM, initially at 0.021, increased to 0.029 when employing its variant ALSTM, along with improvements in all other metrics assessing predictive performance. Specifically, the ICIR of LSTM improved from 0.031 to 0.042 with ALSTM, the rank IC of LSTM rises from 0.016 to 0.026 with ALSTM, and the rank ICIR of LSTM increased from 0.026 to 0.041 with ALSTM. Regarding backtesting performance, as presented in Table 11, the enhancements observed with ALSTM align with the improvements noted in the predictive performance of ALSTM. More specifically, the C-MVO information ratio of LSTM, initially at 0.671, increases to 0.744 with ALSTM; the long-short strategy information ratio of LSTM, initially at 0.168, escalates to 0.579 when implementing ALSTM. These results are possibly due to the attention layer incorporated in ALSTM, which provides ALSTM with an enhanced capability to identify correlations between *VIX*_1_ ∼ *VIX*_6_. However, GRU showed a relatively poor predictive performance, as indicated by an IC value of 0.004. Its backtesting performance was acceptable, with a C-MVO information ratio of 0.241 and–ng a short information ratio of 0.409. However, these metrics fall short of the superior performance of ALSTM. Therefore, within the category of neural-network-based models, ALSTM emerges as the most promising model for predicting the next-day returns of VIX CMFs, particularly when utilizing term-structure feature sets.

#### 5.4.2 Tree-based models

From the perspective of tree-based models, there is variation in the predictive and backtesting performance of XGBoost and LightGBM. In terms of the predictive performance, the IC of XGBoost, initially at 0.047, decreased to 0.035 when the LightGBM framework was used, and the ICIR of XGBoost decreased from 0.071 to 0.066. Conversely, LightGBM outperformed XGBoost in terms of both the rank IC and rank ICIR metrics. In backtesting performance, under the benchmark long-short strategy framework, XGBoost demonstrates a decent information ratio of 0.632 and an annualized return of 0.116, which is acceptable relative to LightGBM, whose metrics are negative across all evaluation metrics. However, under the C-MVO strategy framework, the backtesting results are the opposite of those observed in the long-short strategy framework. Based on the above analysis, the predictive abilities of XGBoost and LightGBM did not correlate with their subsequent backtesting performance. Consequently, to effectively predict the next-day returns of VIX CMFs and utilize these predictions for profitable trading, researchers and practitioners should carefully assess these two models, considering both predictive and backtesting performance simultaneously, to select the most appropriate tree-based model for trading VIX CMFs.

#### 5.4.3 Vanilla models

Among the vanilla models, an intriguing observation is that the linear regression model outperforms the MLP and all other machine learning models. The MLP, categorized as a relatively simple machine learning model, integrates nonlinear relationships through its activation function. However, both in terms of predictive and backtesting performance, the MLP falls short of the linear regression model. This suggests that for predicting the next-day returns of VIX CMFs using term structure features, models focusing on linear relationships might be superior to those that consider more complex nonlinear relationships. This observation further indicates the significant predictive ability of the term structure features in forecasting the next-day returns of VIX CMFs.

#### 5.4.4 Predictive ability of machine learning models

Among the three types of models–RNN-based models, tree-based models, and traditional vanilla models–we found that the linear model outperformed all other models. This could be attributed to the inclusion of sufficiently predictive factors in the term structure feature sets and the prediction of outcomes being inherently linear. Therefore, all nonlinear machine learning models tend to produce poorer predictive results for this forecasting problem owing to overfitting. Models with structures that surpass the intrinsic predictive mechanisms of the dataset may lead to overfitting of samples. This is because the model captures non-existent causal relationships during training.

### 6. Limitations

In our experiment, we employed various innovative and complex models, such as transformers, graph attention networks, ADD, and tabnet. However, the dataset spans from December 20, 2005, to August 15, 2022, comprising a total of 4,022 samples. An insufficient sample size can lead to overly complex model underfitting within the training set. Taking the transformer as an example, we employed an extremely small hidden layer of 64 and a 2-headed attention encoder for training. In this model, the number of parameters of the self-attention block alone was 32768, resulting in poor model fitting, as evidenced by a relatively high training loss. However, models with structures that surpass the intrinsic predictive mechanisms of the dataset may lead to overfitting of the sample. This occurs because the model captures non-existent causal relationships during training. These dual factors contribute to the weak out-of-sample predictive performance of the model. Therefore, we decided not to include these models in the main part of our study; the related performances are included in the S1–S8 Figs.

### 7. Conclusion

This study explores the predictive ability of VIX CMFs term structure features on the next-day returns of VIX CMFs using advanced machine learning models. Based on the numerical predictions of machine learning models, we employ our newly proposed C-MVO strategy, which considers the predicted returns and additional constraints, along with a benchmark rank-based long-short strategy. Both the predictive and backtesting performances are rigorously evaluated using diverse metrics with a strict walk-forward expanding time window procedure, which allows us to avoid future information leakage.

The experiment results indicate that VIX CMFs term structure features, specifically, *μ*_*t*_ and △*roll*, are highly effective in predicting the next-day returns of VIX CMFs and could potentially yield significant economic benefits. However, statistically derived features possess comparatively less predictive ability. Additionally, the C-MVO strategy shows overall superior backtesting performance across most machine learning models compared to the benchmark rank-based long-short strategy, providing valuable insights and practical implications for the formulation of trading strategies involving VIX CMFs and proving that numerically predicted returns can better guide trading strategies. Finally, evaluations of the machine learning models revealed that within the neural-network-based models, ALSTM exhibited the best performance in both predictive and backtesting assessments. No single tree-based model demonstrated clear superiority. More importantly, the linear regression model, which considers only linear relationships, outperforms all other models, thereby affirming the substantial ability of term structure features in predicting the next-day returns of VIX CMFs.

Overall, this study provides a comprehensive survey for developing VIX CMFs trading strategies using machine-learning model numerical predictions for both researchers and practitioners. However, it faces limitations related to the dataset size, which affects the performance of advanced models. Future studies could explore the use of high-frequency data to enlarge the dataset, potentially enhancing the evaluation of advanced machine learning models by mitigating data size constraints.

## Supporting information

S1 Fig RankIC values for all models. (TIF)

S2 Fig Long–short information ratios in all models. (TIF)

S3 Fig C-MVO information ratios for all models. (TIF)

S4 Fig Model netvalues with simple feature set using LongShort strategy. (TIF)

S5 Fig Model netvalues with simple feature sets using C-MVO strategy. (TIF)

S6 Fig Models’ netvalues with termStructure feature sets using LongShort strategy. (TIF)

S7 Fig Model netvalues with derivation feature set using LongShort strategy. (TIF)

S8 Fig Models’ netvalues with feature sets derived using C-MVO strategy. (TIF)
