# Supplementary figures and images for: VIX constant maturity futures trading strategy: A walk-forward machine learning study
Source: PLoS One. 2024 Apr 19;19(4):e0302289. doi: 10.1371/journal.pone.0302289 (PMC11029606; doi:10.1371/journal.pone.0302289)

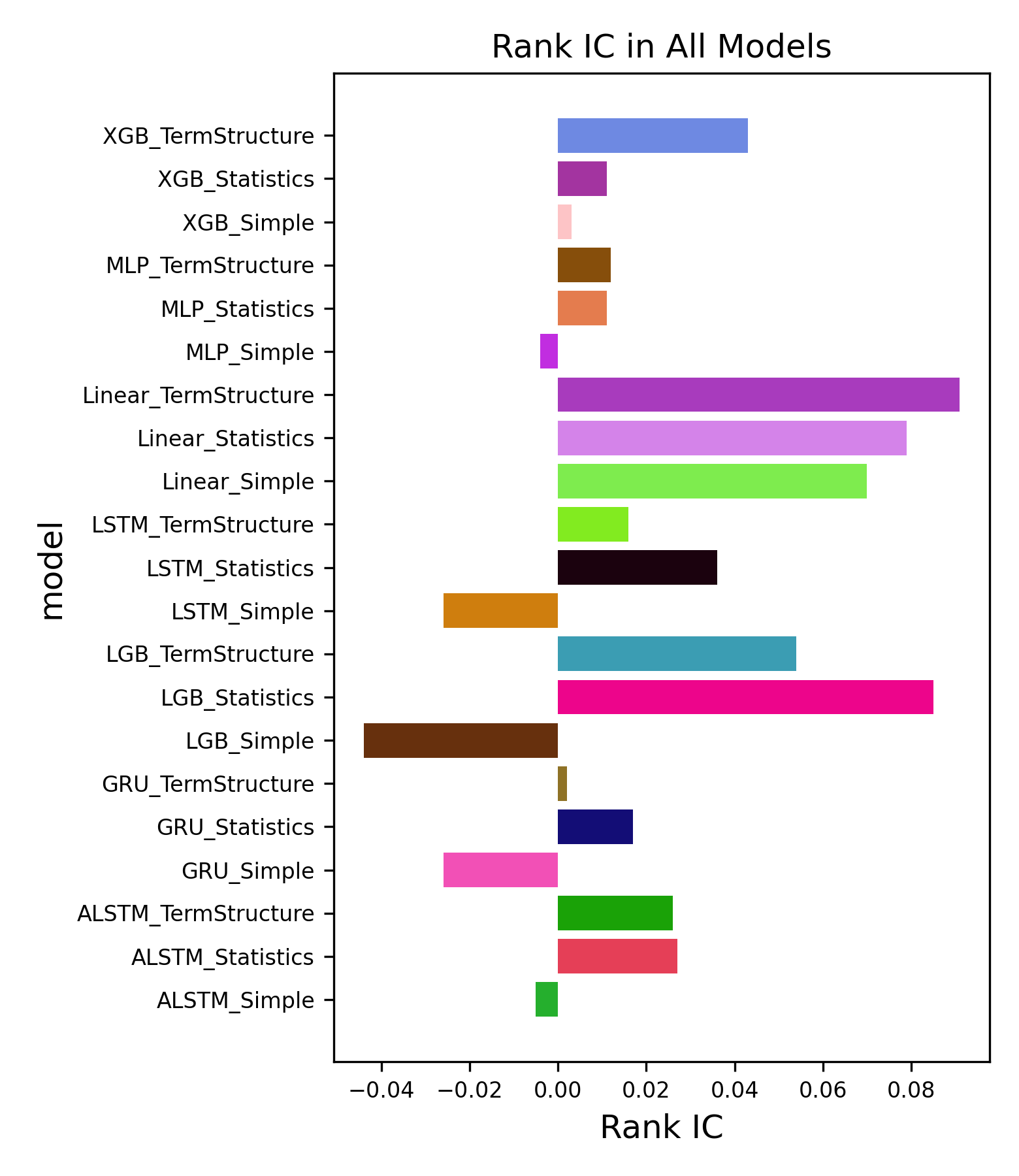

Supplement: S1 Fig — (TIF) [file pone.0302289.s001.tif]

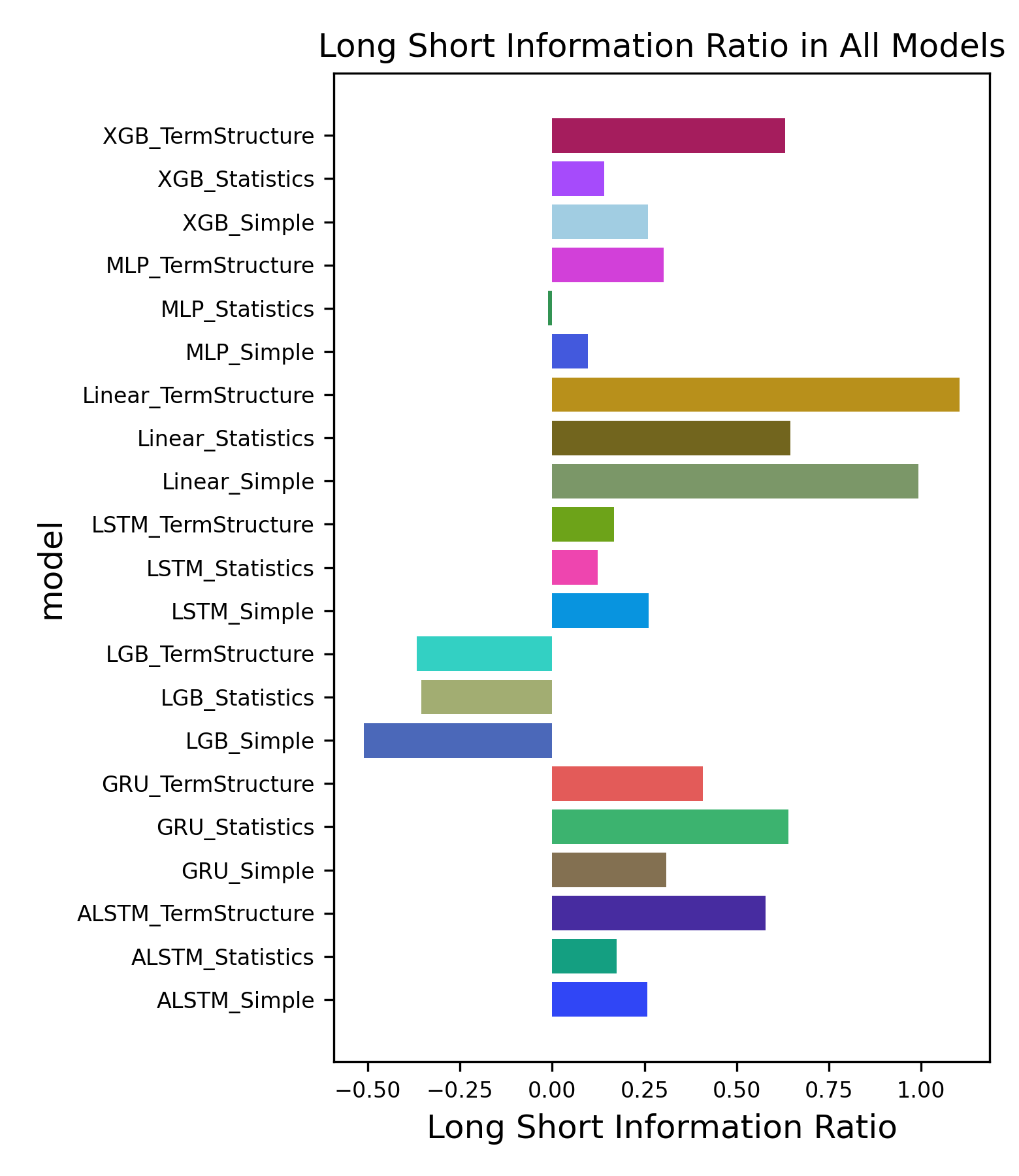

Supplement: S2 Fig — (TIF) [file pone.0302289.s002.tif]

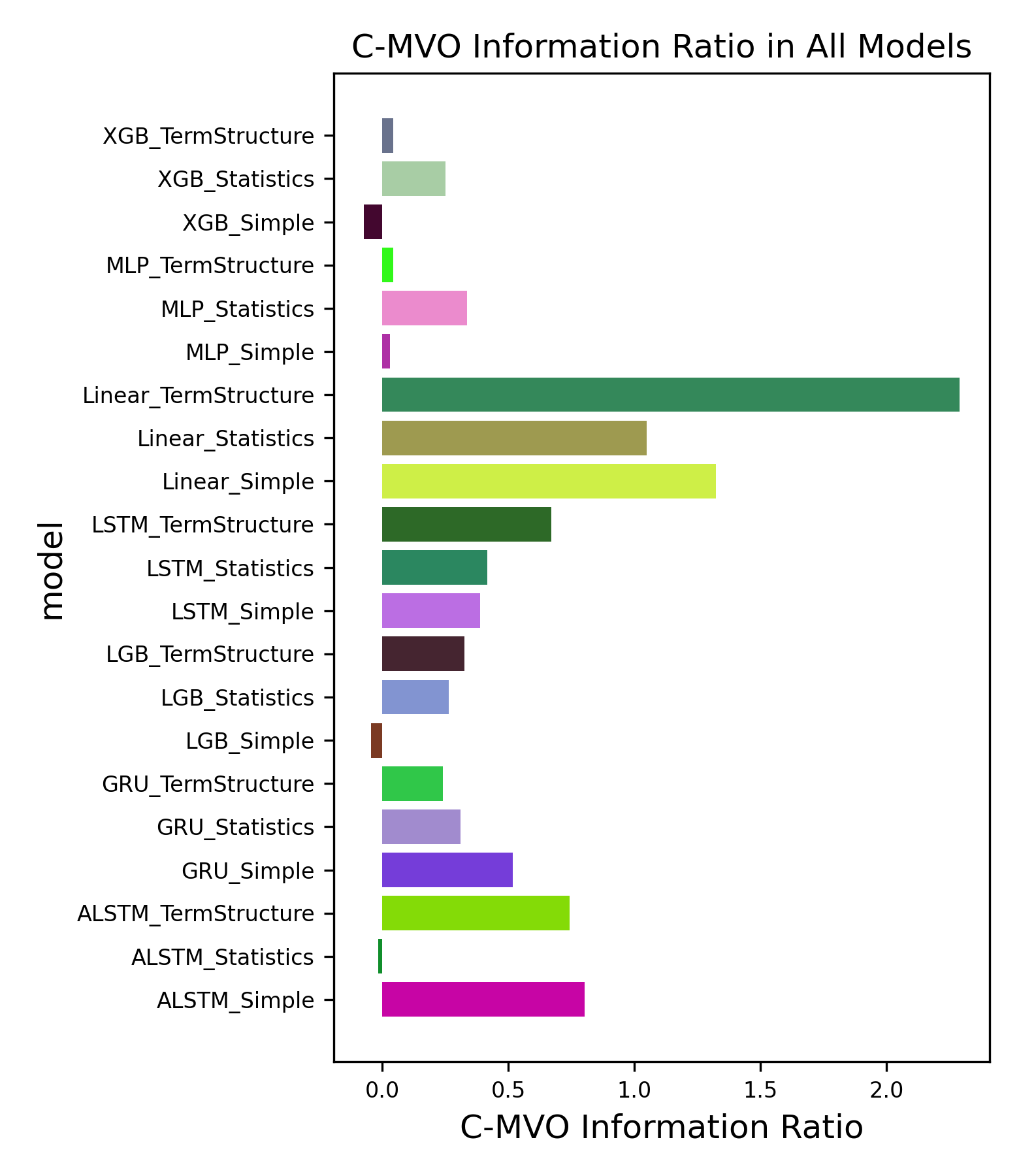

Supplement: S3 Fig — (TIF) [file pone.0302289.s003.tif]

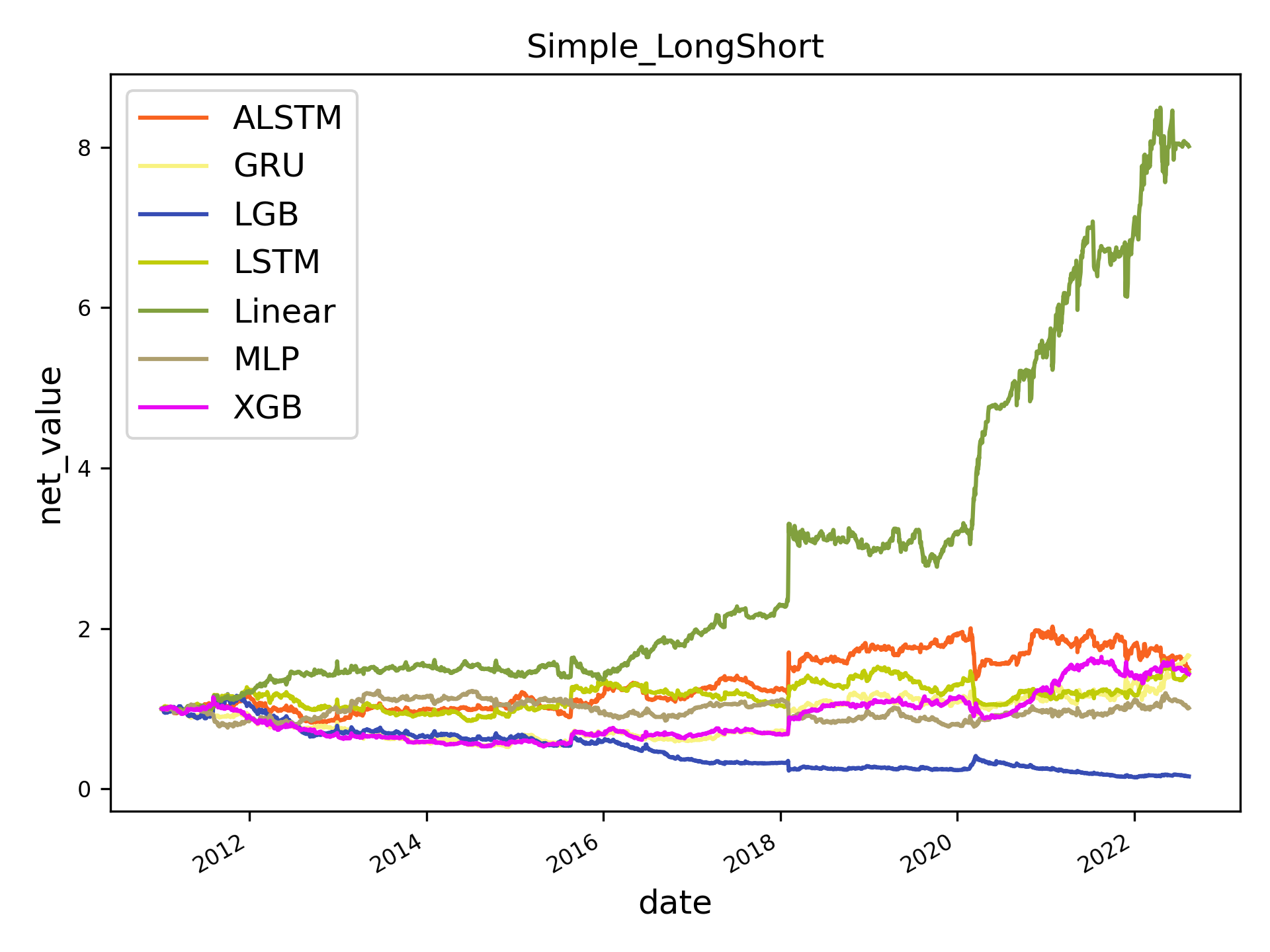

Supplement: S4 Fig — (TIF) [file pone.0302289.s004.tif]

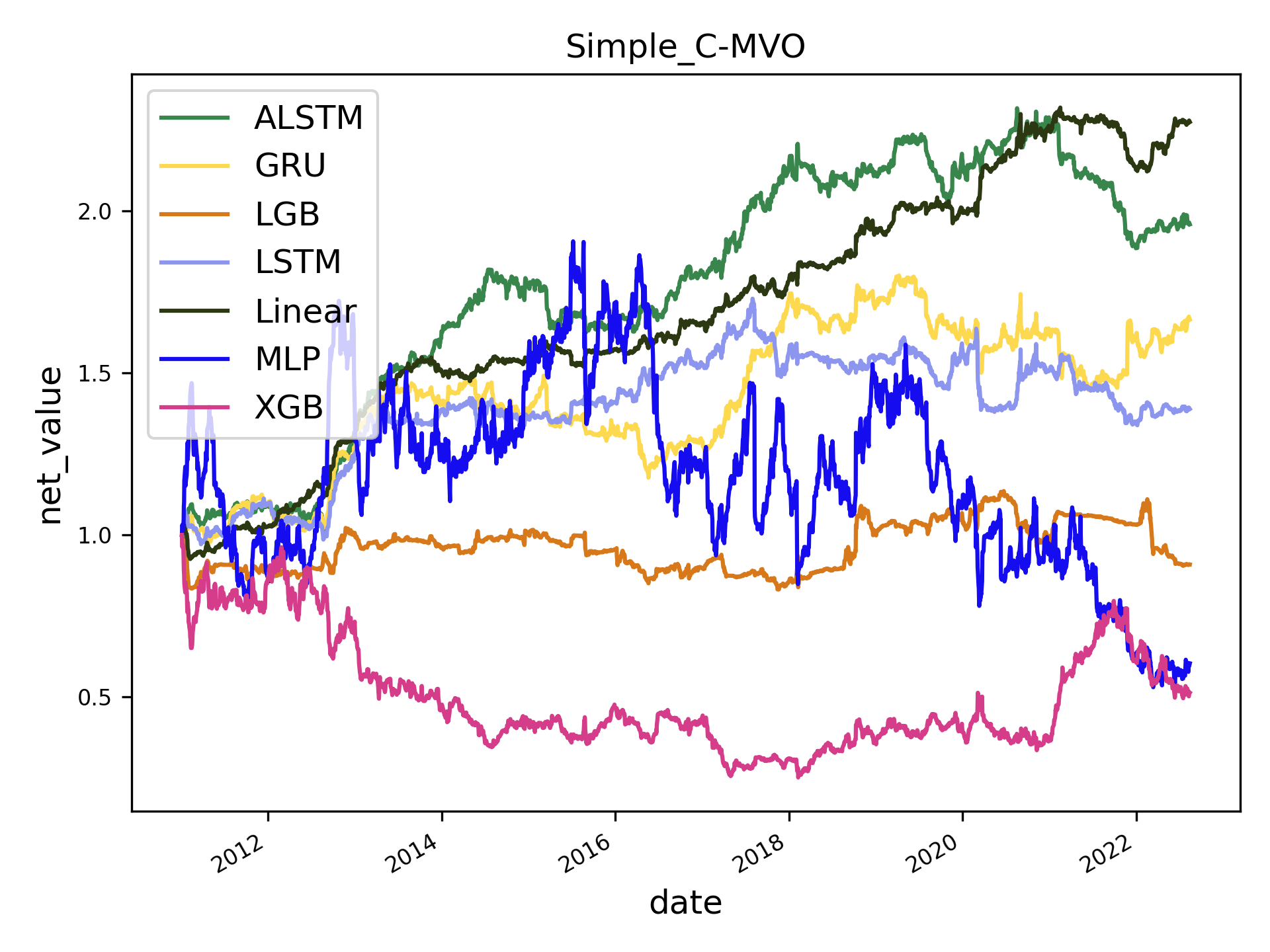

Supplement: S5 Fig — (TIF) [file pone.0302289.s005.tif]

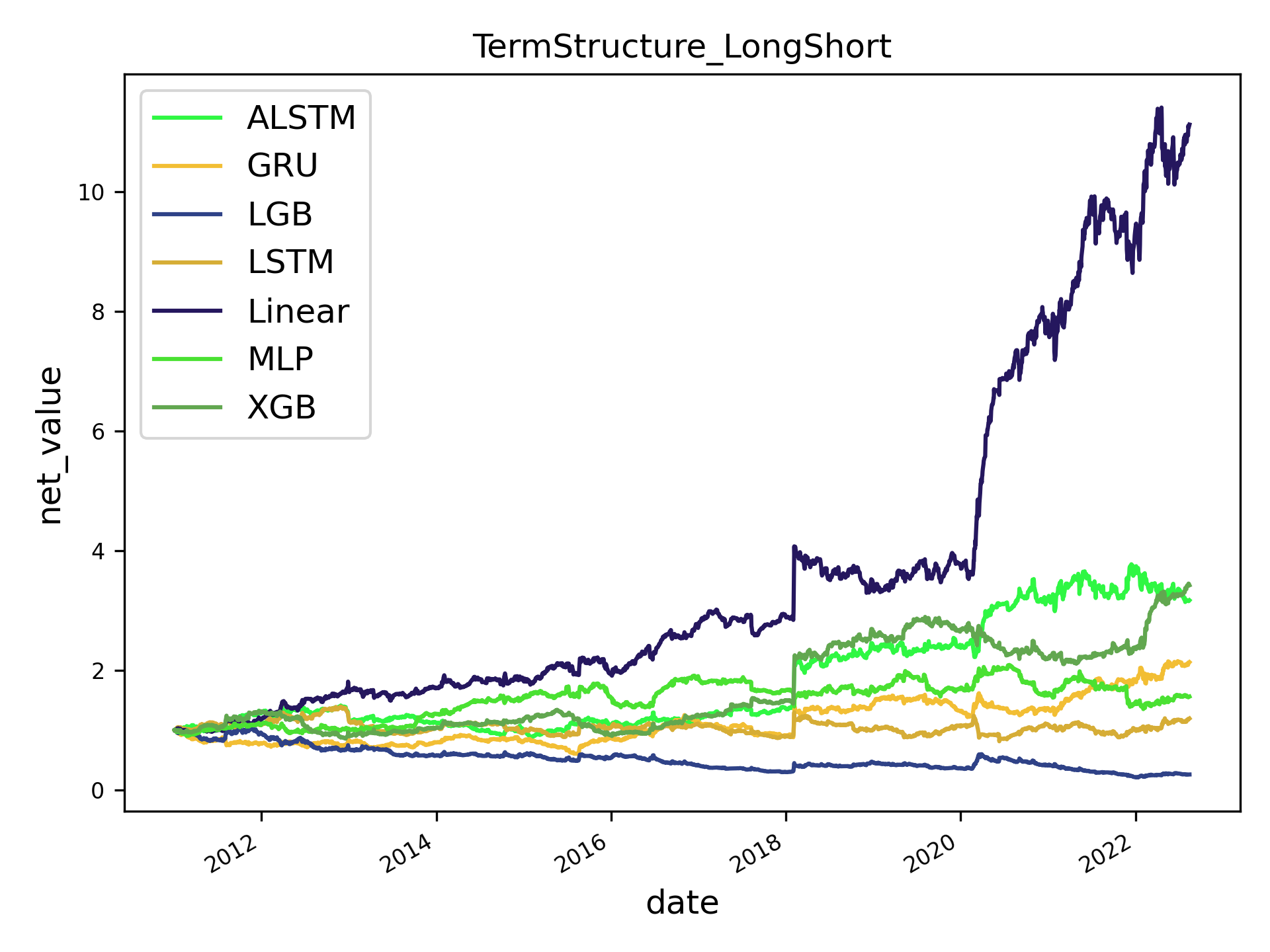

Supplement: S6 Fig — (TIF) [file pone.0302289.s006.tif]

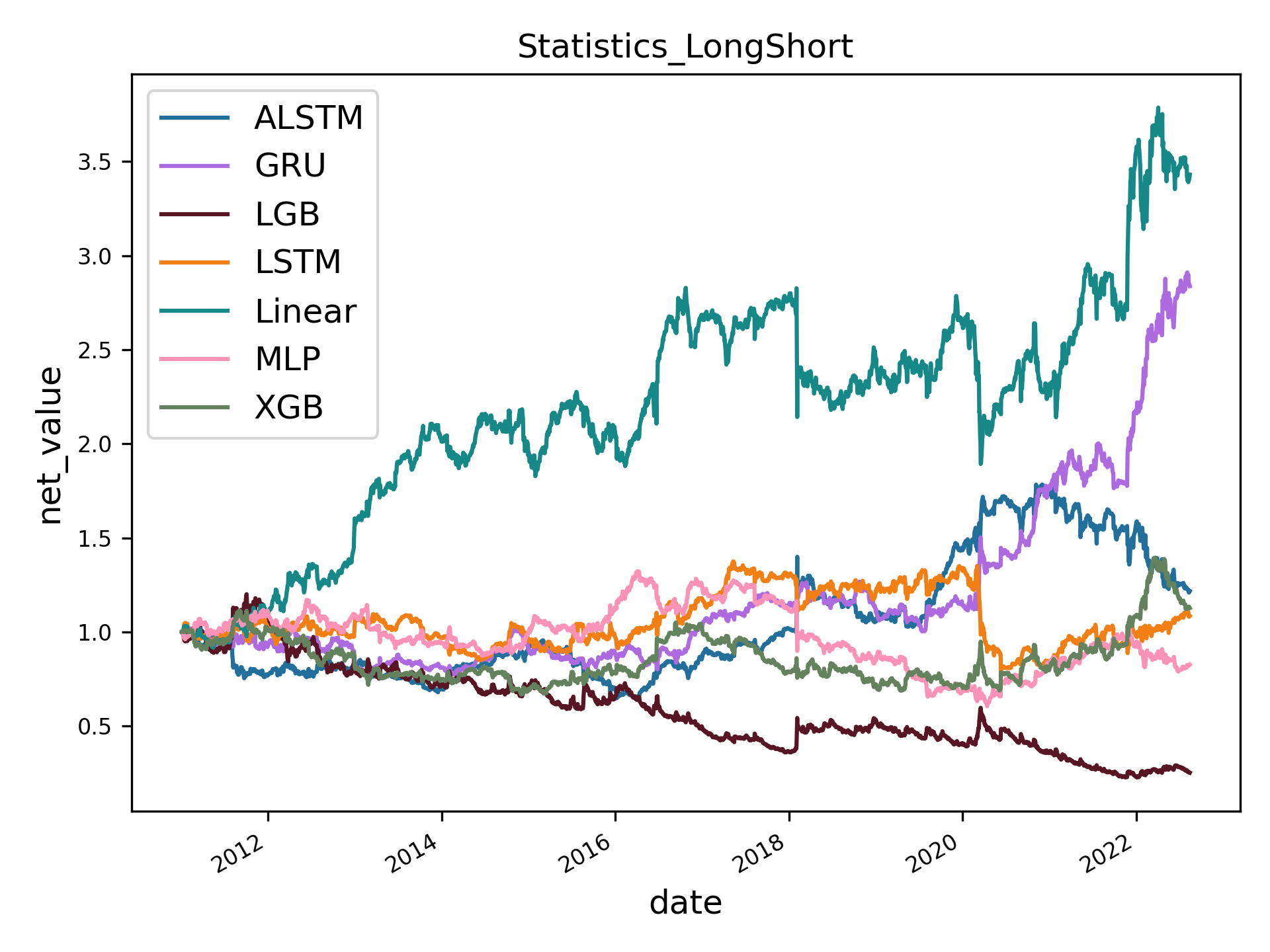

Supplement: S7 Fig — (TIF) [file pone.0302289.s007.tif]

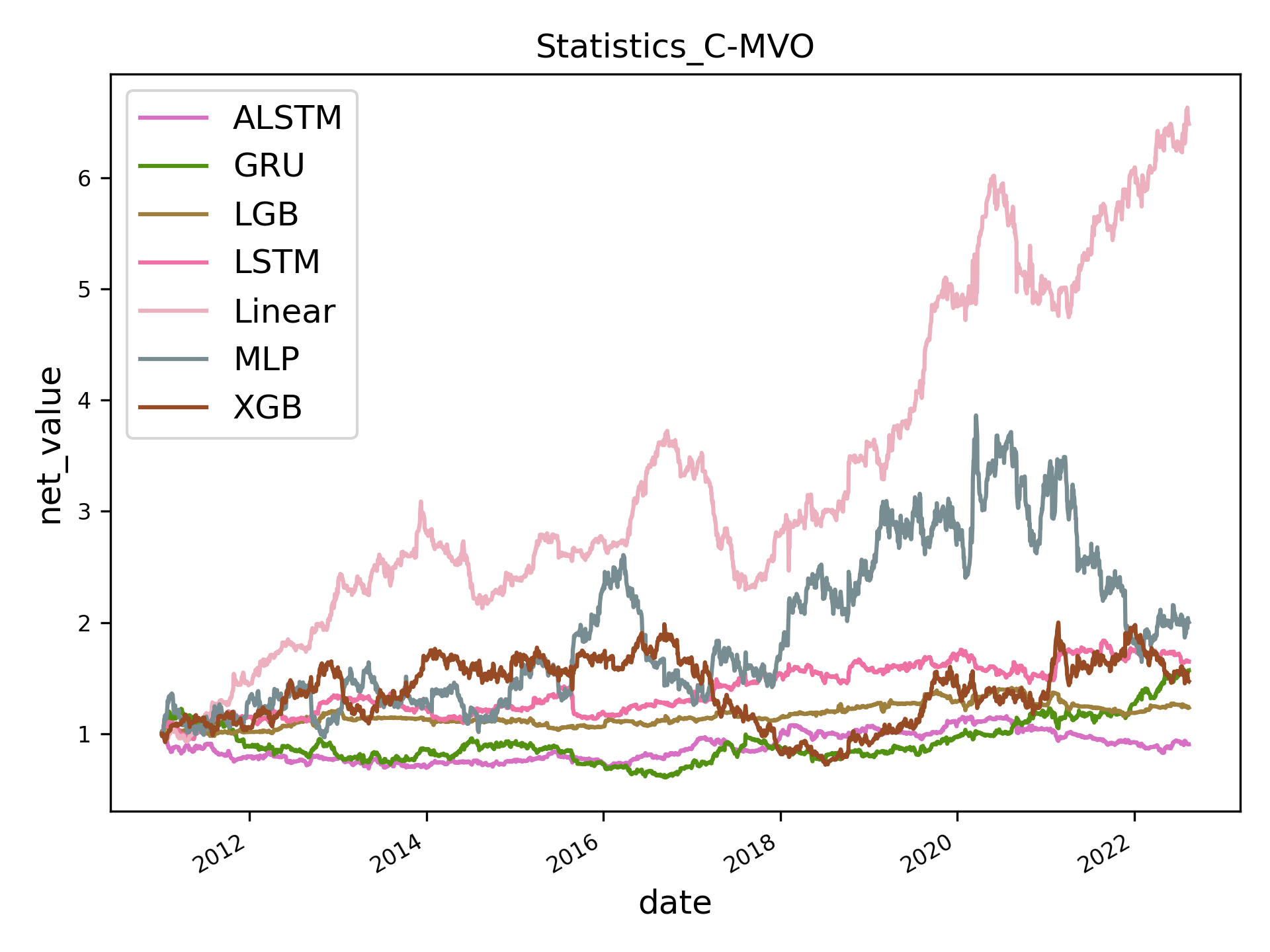

Supplement: S8 Fig — (TIF) [file pone.0302289.s008.tif]
